# A prospective single-arm pilot study evaluating [F-18]fluoroestradiol dedicated breast PET in invasive lobular carcinoma after neoadjuvant endocrine therapy

**DOI:** 10.1186/s13058-026-02277-w

**Published:** 2026-04-11

**Authors:** Kayla M. Switalla, Natsuko Onishi, Ella F. Jones, Courtney Lawhn-Heath, Kimberly M. Ray, Deep K. Hathi, Julia C. Carmona-Bozo, Pouya Metanat, Alexandra J. Lopes, Israel O. Falade, Astrid Quirarte, Soumya Gottipati, Ruby Guo, Julissa Molina-Vega, Kami Pullakhandam, Teffany Joy Bareng, Margarita Watkins, A. Jo Chien, Bonnie N. Joe, Laura J. Esserman, Nola Hylton, Rita A. Mukhtar

**Affiliations:** 1 Department of Surgery, University of California San Francisco, San Francisco, CA USA; 2 Department of Radiology and Biomedical Imaging, University of California, San Francisco, CA USA; 3 School of Medicine, University of California San Francisco, San Francisco, CA USA; 4 Department of Medicine, University of California San Francisco, San Francisco, CA USA

**Keywords:** Dedicated breast PET, ILC, 18F-fluoroestradiol (FES), Neoadjuvant endocrine therapy

## Abstract

**Purpose:**

Invasive lobular carcinoma (ILC) of the breast presents challenges in monitoring response to neoadjuvant endocrine therapy (NET). Dedicated breast positron emission tomography with ^18^F-fluoroestradiol (FES-dbPET), a molecular imaging technique targeting the estrogen receptor (ER), may offer insight into treatment response. This study evaluated whether changes in FES-dbPET uptake before and after NET in patients with ILC correlate with treatment response indicators, and whether these associations differ by endocrine therapy type.

**Methods:**

This prospective pilot study included patients with stage I-III ER positive, human epidermal growth factor 2 (HER2) negative ILC undergoing NET between 2017 and 2021. FES-dbPET was performed pre- and post-NET. Patients were stratified by type of NET received – aromatase inhibitor (AI) therapy or selective estrogen receptor modulator/degrader (SERM/SERD) therapy. The primary goal was to assess correlation between FES-dbPET parameters and change in tumor Ki-67 staining (%), a validated prognostic endpoint after NET.

**Results:**

We enrolled 19 patients with ER + HER2- ILC, of whom 12 underwent both pre- and post-NET FES-dbPET. Among the AI cohort, changes in SUVpk and SULpk from pre- to post-NET each positively correlated with changes in Ki-67, though these associations did not reach statistical significance (r = 0.74, *p* = 0.256; and r = 0.75, *p* = 0.254, respectively). Conversely, among the SERM/SERD cohort, changes in SUVpk and SULpk strongly and positively correlated with changes in Ki-67 (r = 0.92, *p* = 0.027; and r = 0.92, *p* = 0.028, respectively).

**Conclusion:**

In this pilot study of 19 ER + ILC patients, change in FES uptake on dbPET showed some correlations with tumor response to NET. These findings suggest potential for FES-dbPET to capture tumor biology beyond that of standard imaging tools in predicting therapy response for patients with ILC. Further research with larger samples is needed to refine the role of FES-dbPET in improving neoadjuvant treatment monitoring and surgical planning in ILC patients.

**Supplementary Information:**

The online version contains supplementary material available at 10.1186/s13058-026-02277-w.

## Introduction

Invasive lobular carcinoma (ILC) is the most common special histologic subtype of breast cancer, comprising 10–15% of all breast cancer cases [1, 2]. It is characterized by high rates of hormone receptor positivity and the loss of cell adhesion protein E-cadherin, which contributes to its diffuse growth pattern and clinical behavior distinct from the more common invasive ductal carcinoma, also known as carcinoma of no special type [3, 4]. These features reduce the sensitivity of conventional imaging modalities like mammogram and ultrasound, leading to delayed diagnosis and higher rates of mastectomy, axillary dissections, and positive surgical margins [5–10].

Although breast magnetic resonance imaging (MRI) can improve preoperative detection and staging in ILC, it has not consistently reduced rates of surgical morbidity [5, 11]. Moreover, treatment planning in ILC is further complicated by discordance between advanced clinical but molecularly low-risk features [12]. Neoadjuvant endocrine therapy (NET) is increasingly used in ER-positive breast cancer as a strategy to downstage tumors preoperatively. However, in ILC, its use is limited by the lack of reliable, non-invasive biomarkers to predict or monitor treatment response. Change in Ki-67, a commonly used tissue-based proliferation marker, offers some guidance for treatment selection and reflects prognosis, but its clinical use can be limited by inter-laboratory variability and the need for serial biopsies [13–19].

To address the challenges in imaging ER positive tumors, molecular imaging with [18F]-fluoro-17ß-estradiol (FES)—a radiotracer that binds with high affinity to the estrogen receptor (ER)—has emerged as a promising tool. When combined with high-resolution dedicated breast positron emission tomography (dbPFT), FES-dbPET can provide detailed spatial and quantitative insights into ER expression across tumor and surrounding tissues [20–23]. Given that over 90% of ILC tumors are ER-positive, FES-dbPET presents a targeted, non-invasive approach for evaluating endocrine responsiveness in early-stage ILC [24, 25].

Currently, whole-body FES-PET imaging is typically utilized in the setting of disease progression or metastatic disease, with appropriate use criteria including scenarios such as assessing ER functionality for treatment selection, determining ER status of lesions that cannot be biopsied, or potentially for initial staging in patients with low grade ER + IDC or ILC [26]. While prior studies in the metastatic setting have shown that FES uptake correlates with response to endocrine therapy, its application in early-stage disease, particularly during NET and for ILC, remains underexplored [27]. Importantly, interpreting changes in FES uptake during NET depends on the mechanism of the endocrine agent. Aromatase inhibitors (AIs) function by reducing systemic estrogen levels, removing the ligand required for ER-driven tumor growth [28]. In this setting, ER would remain intact and FES-avid; therefore, reductions in FES uptake after treatment with an AI may reflect a true treatment response. In contrast, SERM/SERDs act by directly blocking or degrading the ER itself, potentially reducing FES binding even in the absence of significant tumor regression [29, 30]. Thus, in SERM/SERD-treated patients, reductions in FES uptake may reflect effective receptor blockade rather than tumor response, while persistent FES uptake may indicate incomplete receptor blockade or ER-independent tumor biology—both of which may predict suboptimal treatment response. These mechanistic differences suggest that FES-dbPET may offer distinct insights depending on the therapy used. Prior studies have suggested the potential of FES-PET to predict response to NET [31, 32]; however, ILC is underrepresented in these studies. Thus, we aimed to evaluate this notion specifically in ILC patients based on type of therapy. Additionally, while most studies have evaluated whole-body imaging, very little data on FES with dbPET exist.

To investigate the feasibility of utilizing FES-dbPET to evaluate treatment response in those with ILC, we conducted a pilot study of 19 patients with stage I-III ER + ILC undergoing planned NET. We explored how changes in FES uptake metrics on dbPET correlated with treatment response metrics, stratified by endocrine therapy type. Specifically, we considered two hypotheses: 1) In AI-treated patients, reductions in FES-dbPET uptake would correlate with reduced tumor viability, as measured by changes in tumor size, histologic cellularity, and Ki-67 proliferation index; and 2) In SERM/SERD-treated patients, reductions in FES-dbPET uptake would reflect pharmacologic ER blockade; persistent FES uptake would suggest incomplete receptor inhibition and correlate with worse treatment response indicators, as measured by changes in tumor size, histologic cellularity, and Ki-67 proliferation index. By integrating molecular imaging with histopathologic outcomes, we aimed to assess the potential of FES-dbPET as a non-invasive biomarker of response to NET in early-stage ILC.

## Methods

### Study design

We conducted a single-arm, prospective pilot study for patients > 18 years old with biopsy-proven, ER + / human epidermal growth factor-2 (HER2)-negative ILC planning to undergo NET at our institution between 2017 and 2021. ER staining by immunohistochemistry of ≥ 1% was considered positive. Pregnant or lactating women were excluded. The study was approved by our institution’s Institutional Review Board (IRB#: 16-19959), and patients consented to undergo pre-treatment (“pre-NET”) and post-treatment (“post-NET”) FES-dbPET imaging as part of the study protocol. Of note, FES was prepared at the UCSF Radiopharmaceutical Facility Cyclotron under an Investigational New Drug submission obtained by UCSF (#136929**)** until the year 2020, at which point it became commercially available.

NET was either given as a standard of care clinical management or on other approved clinical trial protocols (e.g. Translational Breast Cancer Research Consortium 037 [TBCR037 Clinical Trial]) and the Endocrine Optimization Protocol of the I-SPY2 Trial) [33]. Patients on any NET were included (selective estrogen receptor modulators [SERM], selective estrogen receptor degraders [SERD], or aromatase inhibitors [AI]. FES-dbPET imaging results were not utilized for clinical management decisions. Accordingly, FES-dbPET was studied as an integrated biomarker in patients who otherwise were undergoing NET. Undergoing breast MRI was not mandated as part of this study, but when breast MRI was obtained as part of clinical care, images were reviewed and results recorded.

Given the pilot nature of this study, the sample size was based on feasibility and funding constraints with the goal of obtaining estimates and confidence intervals to aid with powering future, larger trials. The COVID-19 pandemic coincided with much of the study, which resulted in difficulties with reduced in-person visits, changes in treatment plans, including delays to surgery, and some patients undergoing part of their treatment locally instead of at UCSF. As enrollment and follow-up scans became more challenging, study accrual was stopped in 2021.

### Patient and tumor characteristics

Medical record review was conducted to document patient and tumor characteristics, including age, menopausal status at diagnosis, type and duration of NET, and tumor receptor subtype (ER status, progesterone receptor [PR] status, and HER2 amplification status).

For patients who underwent breast MRI, the longest tumor diameter was extracted from pre- and post-treatment clinical reports. We evaluated longest tumor diameter on MRI instead of volumetric measurements, like functional tumor volume, since longest tumor diameter is used clinically for surgical planning. Additionally, Ki-67 indices from pre-treatment core biopsies and both Ki-67 and tumor cellularity from post-treatment surgical specimens were extracted from pathology reports and evaluated as continuous variables.

### Dedicated breast PET imaging protocol

Baseline dbPET was performed after intravenous injection of FES (5 mCi ± 10%). Of note, although whole-body PET imaging was not a component of this study, our program typically utilizes the same tracer dosing for dbPET protocols as whole-body PET protocols to facilitate patients potentially undergoing both imaging scans on the same day utilizing a single dose of tracer. Using the Mammi platform (Oncovision, Valencia, Spain; Food and Drug Administration clearance in 2016), patients were scanned in the prone position at 45 min after tracer injection with a single breast positioned through the aperture inside the detector ring. Both ipsilateral and contralateral breast were scanned during the same visit, with each breast scanned at 3 min/frame for total of 12–15 min. Follow-up dbPET scans were performed using the same imaging procedure and tracer dose as performed at baseline.

Analyses of tumor FES uptake via dbPET were performed pre-NET and post-NET. FES-dbPET images were reconstructed in 3D using the manufacturer-provided maximum likelihood expectation maximization algorithm with 16 iterations. All images were corrected for attenuation through image segmentation, scatter, and decay. We performed semi-automated segmentation of the tumor volume of interest (VOI) encompassing the abnormal tissue volume using an SUV threshold of 2.5 (OsiriX, Pixmeo, Switzerland).

Our analyses also included measurements of maximum standardized uptake value (SUVmax), mean SUV (SUVmean), the peak value in a 1 cm^3^ spherical region positioned within the segmented tumor (SUVpk), and background standardized uptake value (using the mean) in the ipsilateral breast (SUVbkgi). Additional measurements included maximum, mean, and peak SUV normalized by lean body mass (SULmax, SULmean, and SULpk, respectively) [34]. Measurements were obtained by placing VOIs on tumor and normal breast tissue within the field of view. Tumor uptake volume (TUV), which measures the volume of tumor capable of binding with estrogen, was calculated by summing the SUV of all voxels within the tumor VOI. A TUV of zero indicates the absence of estrogen-binding cells in the tumor, although the tumor itself may still be present despite none or minimal FES TUV.

All dbPET scans were reviewed and analyzed by a nuclear medicine radiologist (EFJ, CLH) to evaluate the uptake of FES. When no tumor could be visually perceived on dbPET, TUV was deemed zero. Reasons for any delays or omissions in obtaining the FES-dbPET scans were documented to assess the feasibility of obtaining pre- and post-NET FES-dbPET.

### Statistical analysis

Baseline patient demographics, clinical stage, and tumor characteristics, including receptor subtype and grade, were recorded for all patients. Patients were stratified by type of NET received: AI or SERM/SERD therapy. Student’s t-tests and chi-squared tests were used to compare baseline characteristics between treatment groups. Tumor size (*i.e. *longest tumor diameter on MRI) and tumor Ki-67 proliferation index were also obtained for all patients at baseline and post-NET when available. These measures were compared between treatment cohorts using student’s t-tests.

Comprehensive data on FES-dbPET timing, NET duration, and FES-dbPET uptake metrics were collected for all patients. Descriptive statistics summarized pre- and post-NET FES-dbPET values, and differences between treatment cohorts were assessed via student’s t-tests. Cases of non-visualization of the tumor on dbPET were reviewed with a nuclear medicine radiologist (CLH) to determine likely causes.

Finally, patient-level data were collected on dbPET parameters and treatment response indicators. For each patient, changes in FES uptake were evaluated by calculating the percent change in predefined uptake metrics between pre- and post-NET dbPET scans. These changes were correlated with post-treatment Ki-67, tumor cellularity, and tumor size using pairwise correlations with Pearson’s coefficients. Scatter plots were generated to visualize the data, and linear fit lines with 95% confidence intervals were overlaid to illustrate the direction and strength of the correlation.

All statistical analyses were performed using STATA 18 (Stata Corp., College Station, TX, USA). Two-tailed *P* values < 0.05 were considered significant.

## Results

### Study cohort characteristics

We obtained FES-dbPET scans for 19 patients with ER + /HER2- ILC for whom NET was planned as pre-operative systemic therapy. One patient included in this study was also included in a prior publication describing the potential role of FES-dbPET for patients undergoing neoadjuvant therapy [34]. Additionally, one patient initially received two cycles of a SERD before ultimately switching to chemotherapy for the remaining five months of neoadjuvant treatment; this patient was excluded from analysis.

Of the remaining 18 patients, the average age was 58 years, ranging from 32–82 years, and 66.7% were post-menopausal at diagnosis. Pre-treatment tumor grade was 2 in the majority (72.2%) of patients, and 50% had stage 2 disease (Table 1).

**Table 1:** Patient baseline demographics and tumor characteristics

	Total (N = 18)	SERM/SERD (n = 7)	AI (n = 8)	No NET (n = 3)
**Demographics**				
Age (years)	58.2	52.1	63.5	54.0
*Menopausal status at diagnosis*				
Pre-menopausal	6 (33.3%)	4 (57.1%)	1 (12.5%)	1 (33.3%)
Post-menopausal	12 (66.7%)	3 (42.9%)	7 (87.5%)	2 (66.7%)
**Baseline tumor characteristics**				
*Tumor grade*				
1	3 (16.7%)	0 (0.0%)	3 (37.5%)	0 (0%)
2	13 (72.2%)	7 (100.0%)	3 (37.5%)	3 (100%)
3	2 (11.1%)	0 (0.0%)	2 (25.0%)	0 (0%)
*Overall stage*				
1	6 (33.3%)	3 (42.9%)	2 (25.0%)	1 (66.7%)
2	9 (50.0%)	4 (57.1%)	3 (37.5%)	2 (33.3%)
3	3 (16.7%)	0 (0.0%)	3 (37.5%)	0 (0%)
4	0 (0.0%)	0 (0.0%)	0 (0.0%)	0 (0.0%)
MRI longest diameter (cm)^b^	4.3 (2.5)	4.8 (2.5)	4.8 (2.4)	2.3 (2.6)
Ki-67 (%)^c^	10 (5–30)	10 (5–20)	10 (5–30)	15 (10–20)

Data expressed as mean (SD), n (%), or median (range)

^a^Stage determined per American Joint Commission on Cancer staging manual edition 7

^b^Data available in 16 patients

^c^Data available in 17 patients

SERM: selective estrogen receptor modulator, SERD: selective estrogen receptor degrader, AI: aromatase inhibitor, ER: estrogen receptor, HER2: human epidermal growth factor receptor 2, NET: neoadjuvant endocrine therapy

Initial treatment received was SERM in one patient (5.3%), SERD in six patients (33.3%), AI in eight patients (44.4%), and three patients (16.7%) proceeded to surgery without NET. Of note, one patient receiving a SERD was concurrently receiving an AI as part of a randomized clinical trial; for purposes of analysis, this patient was included in the SERM/SERD cohort. Additionally, one patient in the AI cohort had last received a SERM six weeks before their baseline FES-dbPET, then subsequently received 29 weeks of AI therapy prior to their post-NET FES-dbPET. This patient was included in the AI cohort due to sufficient washout time from the SERM before the baseline scan [35].

In comparing patients in the SERM/SERD cohort (n = 7) to those in the AI cohort (n = 8), there was a trend towards younger age and more pre-menopausal patients in the SERM/SERD cohort, but this did not reach statistical significance, with baseline age being 52.1 years versus 63.5 years, *p* = 0.14 respectively. However, the cohorts did differ in pre-treatment tumor grade, with the SERM/SERD cohort having a larger proportion of grade 2 tumors (100.0% versus 37.5%, *p* = 0.038) compared to the AI cohort.

Breast MRI results were available for 16 of 18 cases. Seven (43.8%) had baseline MRI only, and nine (56.3%) had both baseline and post-treatment MRI. On the evaluation of clinical breast MRI, the average MRI-based tumor size (*i.e.* longest tumor diameter) at baseline was 4.3 cm (ranging from 0.5–8.0 cm). This did not significantly differ by the type of NET received (SERM/SERD, 4.8 cm; AI, 4.8 cm; *p* = 0.99). In the nine patients with post-treatment breast MRI, the average post-NET MRI-based tumor size was 4.6 cm (ranging from 1.6–7.5 cm). This did not significantly differ by type of NET received (SERM/SERD, 3.4 cm; AI, 6.0 cm; *p* = 0.11).

Tumor Ki-67 measurement at one or more time points was available for all 18 cases. Four (22.2%) had pre-treatment Ki-67 only, one (5.6%) had post-treatment Ki-67 only, and 13 (72.2%) had both pre- and post-treatment Ki-67. In the 17 patients with pre-treatment Ki-67 data, the median pre-treatment Ki-67 was 10% (ranging from 5–30%). In the 14 patients with post-treatment Ki-67 data, the median post-treatment Ki-67 was 4% (ranging from 1–20%).

### Feasibility of study procedures

Of the 18 included patients, 12 (66.7%) underwent both pre- and post-NET FES-dbPET (after a range of 2–41 weeks of NET), five (27.8%) underwent only pre-NET FES-dbPET (of which three patients proceeded directly to surgical resection without undergoing NET), and one (5.6%) underwent only post-NET FES-dbPET. The patients who proceeded to surgical resection without undergoing any NET ultimately had a change in their management preference. Of those who underwent any NET (n = 15), FES-dbPET was delayed at baseline timepoint in three patients, with these patients undergoing imaging from 8–87 days after starting NET. Reasons for delays were logistical in nature and included patients being unavailable for the appointment or the FES tracer batch not meeting quality control requirements; this occurred before the commercial availability of the tracer.

### Baseline FES-dbPET parameters

Of all patients with pre-NET FES-dbPET (n = 17), primary tumor uptake was distinguishable from the background normal tissue uptake in 15 patients (88.2%) with an overall average TUV of 9.7 cm^3^. Reasons for non-visualization of the primary tumor on baseline FES-dbPET scan included uncertainty regarding whether the tumor was within the dbPET field of view and lack of concordance between dbPET and MRI findings. Background SUV in the ipsilateral breast ranged from 0.5–2.8. The SERM/SERD cohort exhibited an average pre-NET TUV of 13.2 cm^3^, while the AI cohort had a statistically similar average pre-NET TUV of 9.5 cm^3^ (*p* = 0.74). Baseline SUVmax and SUVmean were similar in the two cohorts (SUVmax 16.3 (standard deviation [SD] 6.1) in SERM/SERD cohort and 15.5 (SD 8.1) in AI cohort; SUV mean 6 (SD 2.1) in SERM/SERD cohort and 5.3 (SD 1.4) in AI cohort). Additional baseline FES-dbPET measurements are shown in Table 2.

**Table 2:** Baseline and post-treatment FES-dbPET measurements in patients who underwent neoadjuvant endocrine therapy with both pre-treatment and post-treatment FES-dbPET imaging

FES-dbPET parameters	Total (n = 12)		SERM/SERD cohort (n = 6)		AI cohort (n = 6)	
	Pre-treatment (baseline)	Post-treatment	Pre-treatment (baseline)	Post-treatment	Pre-treatment (baseline)	Post-treatment*
TUV (cm^3^)	12.4 (18.9)	5.4 (10.9)	13.2 (24.0)	6.7 (14.4)	11.3 (13.2)	4.0 (7.0)
SUVmax^a^	15.9 (6.9)	10.7(9.2)	16.3 (6.1)	9.6 (7.9)	15.5 (8.1)	12.0 (11.3)
SUVmean	5.6 (1.8)	3.4 (1.2)	6.0 (2.1)	3.5 (1.4)	5.3 (1.4)	3.2(1.1)
SUVpeak^a^	4.3 (1.6)	2.3 (1.5)	3.7 (1.5)	1.9 (1.4)	4.8 (1.7)	2.8 (1.7)
SULmax^a^	10.8 (3.8)	7.0 (5.2)	11.5 (3.8)	6.7 (5.0)	10.1 (4.1)	7.3 (6.0)
SULmean	3.9 (1.4)	2.3 (0.8)	4.3 (1.5)	2.5 (1.1)	3.6 (1.2)	2.1 (0.6)
SULpeak^a^	2.9 (1.2)	1.5 (1.0)	2.6 (0.9)	1.3 (0.9)	3.2 (1.3)	1.8 (1.1)

Data expressed as mean (SD)

^a^Post-treatment data available in 5 of 6 patients (subject 21 tumor was small and abutting chest wall; nuclear medicine physician able to determine post-treatment TUV, SUVmean, and SULmean only)

^*^One patient had a posterior primary breast lesion with FES uptake on pre-NET dbPET, but did not demonstrate FES uptake on post-NET dbPET. While it is possible that the post-NET dbPET did not capture the lesion of interest given its posterior location, upon further review it was concluded that the lesion was likely imaged on post-NET dbPET and truly lacked FES uptake (i.e. TUV was considered zero)

FES-dbPET 18F: fluoroestradiol dedicated-breast positron emission tomography, SERM: selective estrogen receptor modulator, SERD: selective estrogen receptor degrader, AI: aromatase inhibitor, NET: neoadjuvant endocrine therapy, TUV: tumor uptake volume, SUV: standard uptake volume, SUL: standard uptake volume normalized by lean body mass

### Post-NET FES-dbPET parameters

**Fig. 1:**
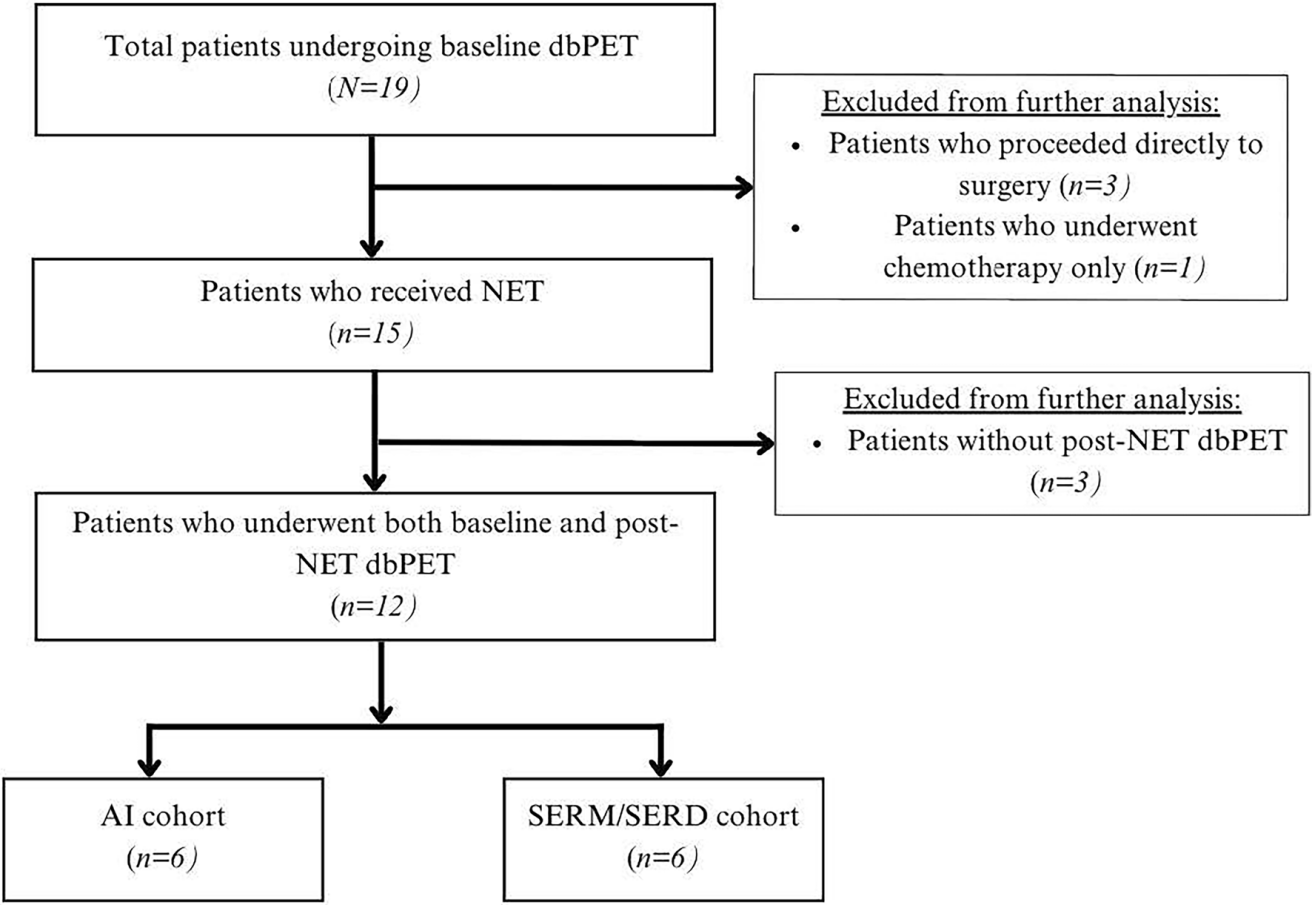
Flow chart showing patients who enrolled in study and analysis cohorts by type of neoadjuvant endocrine therapy. dbPET: dedicated-breast positron emission tomography, NET: neoadjuvant endocrine therapy, AI: aromatase inhibitor, SERM/SERD: selective estrogen receptor modulator/degrader

Out of 18 total patients, 12 had both baseline and post-NET dbPET scans (Fig. 1). Of these patients, primary tumor uptake on post-NET scan was distinguishable from the background normal tissue uptake in nine cases (75.0%) with an average post-NET TUV of 5.4 cm^3^. In two cases, tumors were indistinguishable from background even when utilizing a reference MRI, so FES TUV was deemed zero. In one additional case, the patient had a posterior primary breast lesion with FES uptake on pre-NET dbPET, but did not demonstrate FES uptake on post-NET dbPET. While it is possible that the post-NET dbPET did not capture the lesion of interest given its posterior location, upon further review it was concluded that the lesion was likely imaged on post-NET dbPET and truly lacked FES uptake; thus, TUV was also deemed zero. In one case (subject 18), change in TUV was not calculated due as baseline FES uptake being observed in a region that did not correlate well with tumor on MRI (Table 3). There was no significant difference in average post-treatment TUV between the SERM/SERD and AI cohorts (6.7 cm^3^ vs 4.0 cm^3^, *p* = 0.69). The average decline in FES TUV was similar between SERM/SERD and AI cohorts (61.2% versus 73.6%, *p* = 0.58). There were no statistically significant differences in post-treatment SUVmax or SUVmean between the two cohorts. Post-treatment SUVmax was 9.6 (SD 7.9) in SERM/SERD cohort and 12.0 (SD 11.3) in AI cohort; post-treatment SUV mean was 3.5 (SD 1.4) in SERM/SERD cohort and 3.2 (SD 1.1) in AI cohort). Additional post-treatment FES-dbPET measurements are shown in Table 2.

**Table 3:** Changes in FES-dbPET parameters and tumor response indicators in six patients on AI with both pre- and post-NET dbPET scans. AI, aromatase inhibitor; CDK4/6i, cyclin dependent kinase 4/6 inhibitor

Subject ID	NET	Duration of NET	% decrease TUV	% decrease SUVmax	% decrease SUVmean	% decrease SUVpk	% decrease SULmax	% decrease SULmean	0% decrease SULpk	% decrease Ki-67	% decreasetumor size*	Post-NET cellularity (%)
5	AI (letrozole)	2 months	84.5%	30.6%	16.7%	46.6%	30.9%	17.1%	46.8%	96.7%	− 4.5%	25
13	AI + CDK 4/6i (letrozole + abemaciclib)	4 months	44.6%	− 0.8%	31.2%	33.9%	0.7%	32.2%	34.9%	n/a^a^	1.3%	10
16	AI + CDK 4/6i (exemestane + abemaciclib)	2 weeks	38.7%	16.7%	− 0.3%	− 13.6%	16.0%	− 1.2%	− 14.5%	40.0%	5.6%	50
18	AI (anastrozole)	6 months	n/a^b^	48.5%	40.3%	41.7%	47.0%	36.7%	40.0%	80.0%	n/a^c^	n/a^d^
21	AI (letrozole)	5.5 months	100.0%	n/a^e^	73.1%	n/a^e^	n/a^e^	73.0%	n/a^e^	n/af	n/a^c^	n/a^f^
23	AI (letrozole)	1.5 months	100.0%	70.9%	49.0%	66.9%	70.7%	48.7%	66.7%	70.0%	n/a^c^	95

^*^% change was calculated using pre- and post-NET MRI longest tumor diameter

^a^Pre-NET Ki67 not recorded on pathology report and tissue not available

^b^Pre-NET TUV not calculated out of abundance of caution; tumor uptake on dbPET does not correlate with tumor location on MRI

^c^Post-NET MRI not completed

^d^Cellularity not recorded for surgical pathology specimen and tissue not available

^e^Unable to measure due to small lesion size and abutting chest wall

^f^Declined surgery

### Correlations between changes in FES-dbPET variables and Ki-67, MRI longest tumor diameter, and tumor cellularity

To assess whether changes in FES-dbPET uptake reflect response to endocrine therapy differently by therapy class, we evaluated correlations between imaging metrics and standard indicators of treatment response. Among the AI cohort, changes in FES-dbPET variables did not strongly correlate with changes in Ki-67 (*p* > 0.05), changes in MRI longest tumor diameter (*p* > 0.05), or post-NET tumor cellularity (*p* > 0.05) (Table 3).

**Fig. 2:**
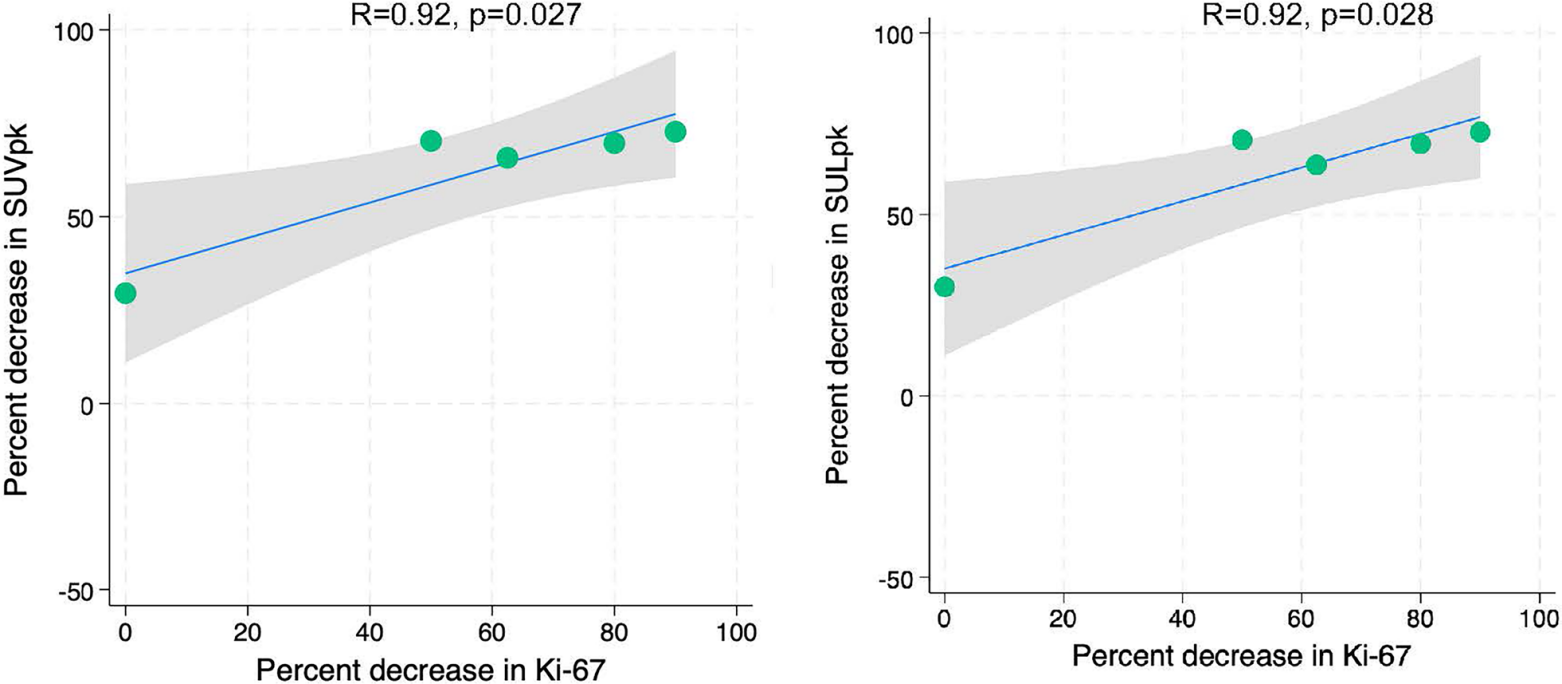
Scatter plots demonstrating correlations between changes in FES-dbPET metrics and change in Ki-67 in the SERM/SERD cohort. Scatter plots utilized linear regression fits and 95% confidence intervals. FES: 18F-fluoroestradiol, dbPET: dedicated-breast PET, SERM: selective estrogen receptor modulator; SERD: selective estrogen receptor degrader; SUVpk: peak standard uptake value; SULpk: peak standard uptake value normalized by lean body mass

**Fig. 3:**
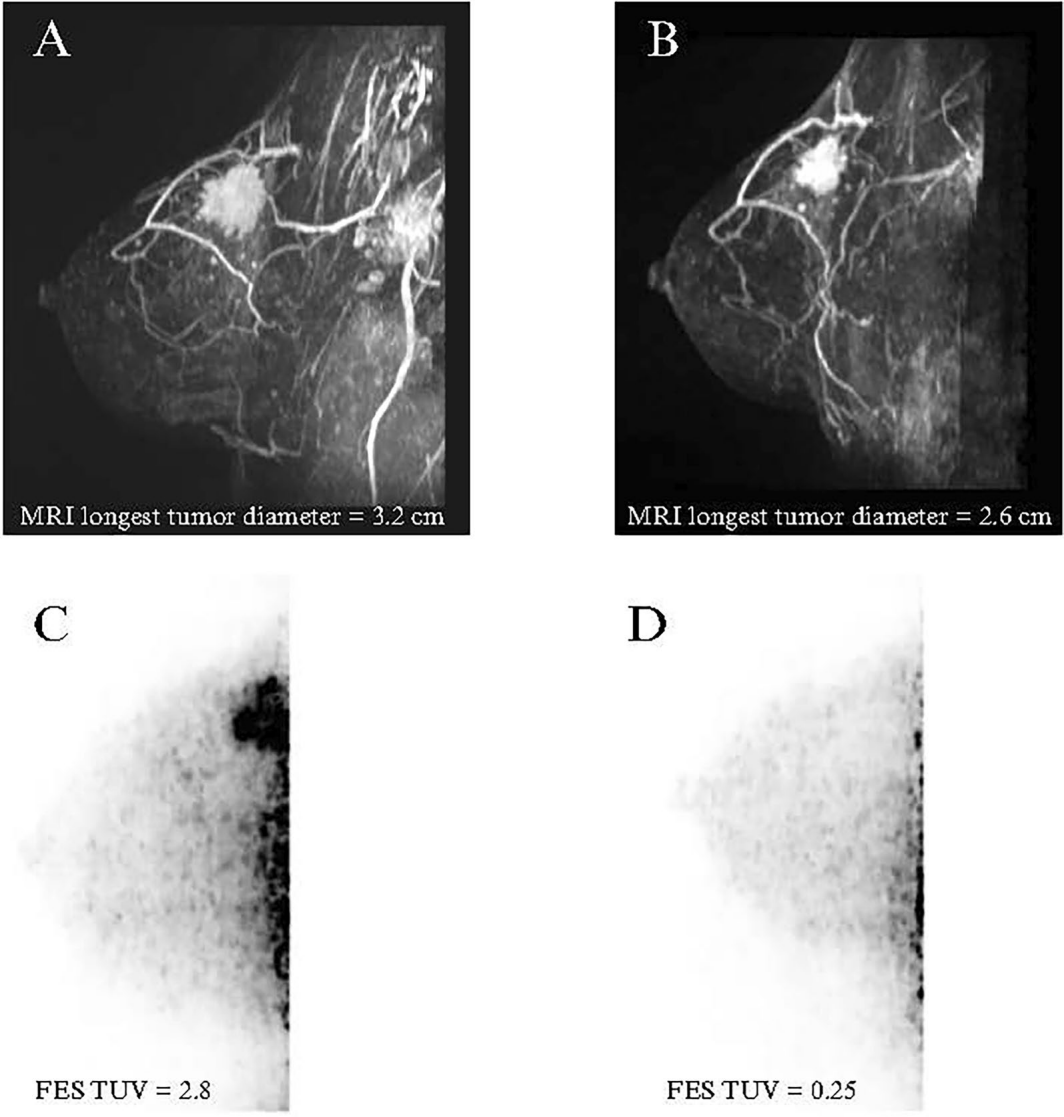
PID 17-images in a 32 year old patient with right breast invasive lobular carcinoma. **A**, **B** Sagittal maximum intensity projection post contrast T1-weighted subtracted 3D MRI scans show minimal change in enhancing tumor volume from (**A**) baseline to (**B**) 24 weeks after initiating neoadjuvant endocrine therapy with a SERM/SERD. **C**, **D** In contrast, sagittal maximum intensity projection dedicated breast PET images show significant decrease in 18F-fluoroestradiol uptake from (**C**) baseline to (**D**) 24 weeks after initiating neoadjuvant endocrine therapy with a SERM/SERD, consistent with ER blockade. SERM: selective estrogen receptor modulator, SERD: selective estrogen receptor degrader

**Fig. 4:**
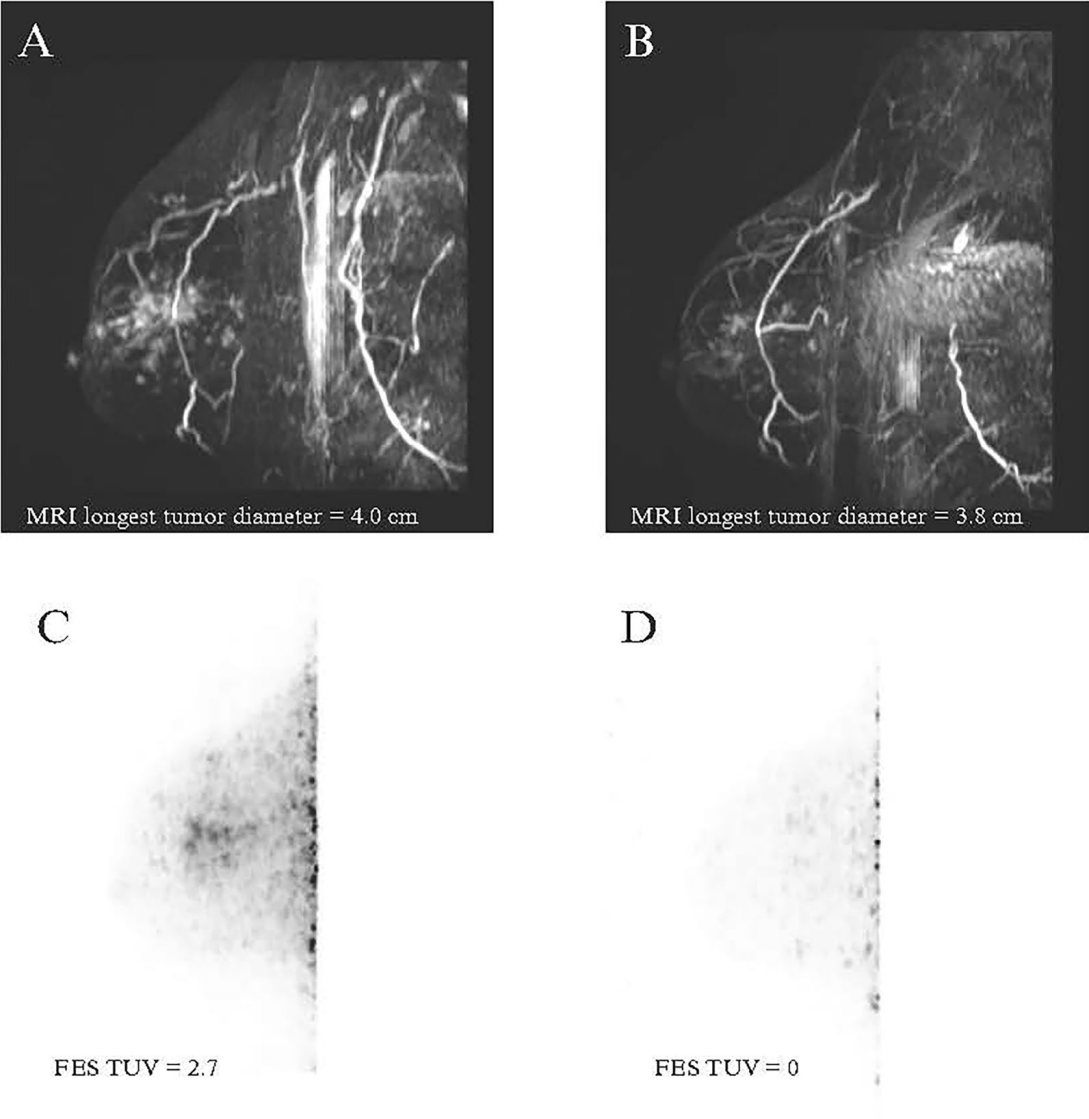
PID 28-images in a 72 year old patient with left breast invasive lobular carcinoma. **A**, **B**, **C** Sagittal maximum intensity projection post contrast T1-weighted subtracted 3D MRI scans show some reduction in enhancing tumor volume but minimal change in longest tumor diameter from (**A**) baseline to (**B**) 13 weeks after initiating neoadjuvant endocrine therapy with a SERM/SERD. In contrast, sagittal maximum intensity projection dedicated breast PET show significant decrease in 18F-fluoroestradiol uptake from (**C**) baseline to (**D**) 4 weeks after initiating neoadjuvant endocrine therapy with a SERM/SERD. SERM: selective estrogen receptor modulator, SERD: selective estrogen receptor degrader

Conversely, among the SERM/SERD cohort, changes in SUVpk and SULpk strongly and positively correlated with changes in Ki-67 (r = 0.92, *p* = 0.027 and r = 0.92, *p* = 0.028, respectively), meaning that a decrease in FES uptake was significantly associated with a decrease in Ki-67 (Fig. 2). SUVpk and SULpk were also positively correlated with change in MRI longest tumor diameter, though these associations did not reach statistical significance (r = 0.81, *p* = 0.098; r = 0.80, *p* = 0.106, respectively). Changes in FES-dbPET variables did not strongly correlate with post-NET tumor cellularity for the SERM/SERD cohort (*p* > 0.05) (Table 4). Examples of pre- and post-NET FES-dbPET are shown in Figs. 3 and 4.

**Table 4:** Changes in FES-dbPET parameters and tumor response indicators in six patients on SERM/SERD with both pre- and post-NET dbPET scans. SERD, selective estrogen receptor degrader; SERM, selective estrogen receptor modulator

Subject ID	NET	Duration of NET	% decrease TUV	% decrease SUVmax	% decrease SUVmean	% decrease SUVpk	% decrease SULmax	% decrease SULmean	%decrease SULpk	%decrease Ki-67	%decrease tumor size*	Post-NET cellularity (%)
14	SERD (fulvestrant)	1 month	41.5%	12.1%	− 0.6%	− 17.6%	14.0%	1.6%	− 15.0%	n/a^a^	n/ab	n/a^a^
17	SERD (amcenestrant)	5.5 months	90.9%	66.8%	58.4%	65.8%	64.7%	55.9%	63.7%	62.5%	18.8%	60
20	SERD (amcenestrant)	5.5 months	− 10.0%	− 14.4%	32.9%	69.7%	− 15.3%	32.4%	69.5%	80.0%	40.0%c	5
24	SERD (fulvestrant)	3 months	69.2%	29.4%	6.5%	29.5%	29.9%	7.2%	30.1%	6.5%	− 23.1%	40
28	SERD (amcenestrant)	5.5 months	100.0%	100.0%	54.8%	70.3%	100.0%	55.3%	70.5%	54.8%	5.0%	20
29	SERD + AI (amcenestrant + letrozole)	5.5 months	75.6%	74.4%	70.3%	72.7%	74.2%	70.3%	72.7%	− 0.3%	14.3%	15

^*^% change was calculated using pre- and post-NET MRI longest tumor diameter

^a^Declined surgery

^b^Post-NET MRI not completed

### Correlation with baseline FES-dbPET and response to therapy

**Fig. 5:**
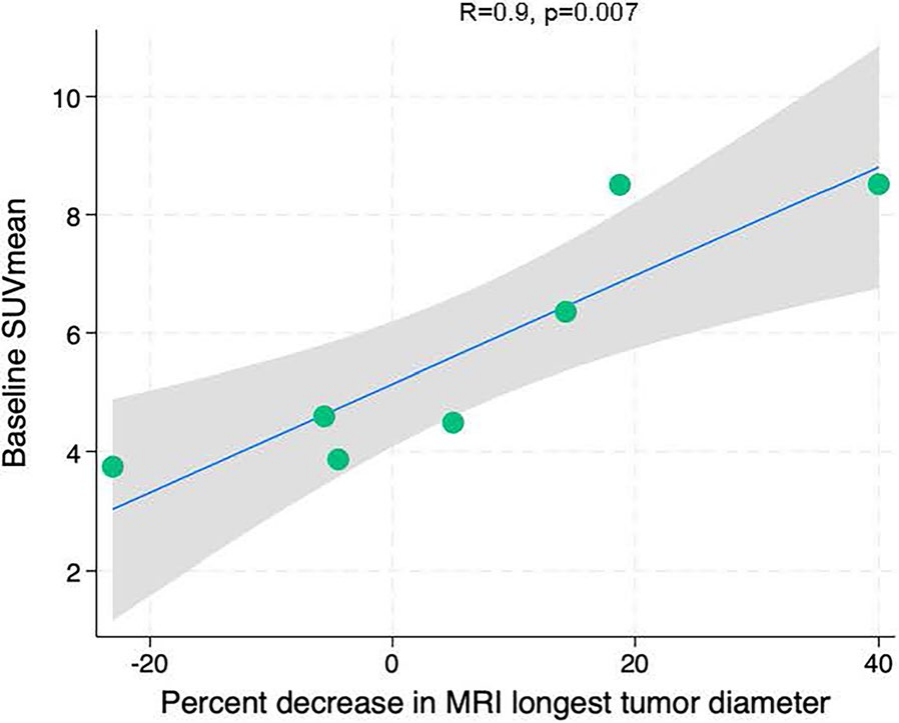
Scatter plot demonstrating correlation between baseline SUVmean and change in MRI longest tumor diameter in the SERM/SERD cohort. Scatter plots utilized linear regression fits and 95% confidence intervals. FES: 18F-fluoroestradiol, dbPET: dedicated-breast PET, SERM: selective estrogen receptor modulator; SERD: selective estrogen receptor degrader; SUVpk: peak standard uptake value; SULpk: peak standard uptake value normalized by lean body mass

Of the 12 patients who had both pre- and post-NET FES-dbPET, nine (75.0%) underwent their baseline FES-dbPET scan prior to starting NET, while three (25.0%) had a delay in their baseline scan. Of the nine patients without delay in baseline FES-dbPET, we evaluated the relationship between FES-dbPET baseline values and response indicators (reduction in Ki-67, reduction in longest diameter on breast MRI, and post-NET tumor cellularity). Pre-treatment FES-dbPET parameters were not significantly correlated with reductions in Ki-67 or with post-NET tumor cellularity. However, there was a significant, positive correlation between baseline SUVmean and reduction in MRI based longest tumor diameter from pre- to post-NET (r = 0.9, *p* = 0.007) (Fig. 5). Additionally, baseline TUV was significantly but negatively correlated with reduction in MRI-based tumor longest diameter (r = 0.8, *p* = 0.027).

## Discussion

The unique characteristics of ILC, such as the absence of E-cadherin and diffuse tumor growth, make it a challenging breast cancer subtype to image and monitor [36]. As NET becomes a promising treatment strategy for early-stage ILC but lacks robust indicators of treatment response, we conducted a prospective pilot study testing the utility of serial dbPET with FES, a surrogate marker for ER expression and positivity, to reflect response to various NET strategies [37]. The ultimate purpose of this pilot study was to determine the feasibility of utilizing dbPET with FES as a non-invasive way to assess ILC response to treatment in the neoadjuvant setting [21, 34, 38]. 

In this prospective pilot study of 19 patients with early-stage ER + ILC undergoing NET, we demonstrated that most enrolled patients were able to complete pre- and post-treatment FES-dbPET with 12 of 19 patients completing paired scans. In the early years of the study, the lack of commercial tracer availability negatively impacted completing study procedures; once the commercial FES tracer was available in the year 2020 this logistical challenge was resolved. However, this coincided with another major barrier to completion of the study incurred by the global pandemic; many visits were changed to telehealth and video appointments, and some patients chose to have surgery locally which led to difficulties with obtaining tissue for analysis. Tying research scans to routinely scheduled clinical visits was a successful strategy in increasingly adherence to the study protocol, but this approach was severely hampered by the pandemic, leading us to stop study accrual.

However, despite the small number of patients who ultimately completed the study, we surprisingly found indications that dbPET with FES may predict response to NET in patients with early-stage ILC, with more pronounced findings in the cohort of patients treated with SERM/SERDs. This is perhaps unexpected, especially since use of SERM/SERD is contraindicated in the setting of whole-body staging with FES due to drug blocking the binding of FES to ER. However, the strategy of using serial FES imaging as an indicator of response to therapy differs from the systemic staging approach, since ongoing FES uptake in the setting of SERM/SERD use may indicate lack of treatment efficacy.

In AI-treated patients, reductions in FES uptake on dbPET parallelled reductions in Ki-67, while persistent FES signal after SERM/SERD therapy, and therefore lack of complete ER suppression, correlated with somewhat attenuated responses in Ki-67 and MRI longest tumor diameter. These data support our hypotheses and highlight the potential of FES-dbPET as a non-invasive biomarker for treatment tailoring in ILC. Additionally, higher baseline SUVmean correlated with greater reduction in longest tumor diameter on MRI, consistent with prior studies showing that FES uptake predicts response to endocrine therapy [39].

### AI cohort: trends in FES uptake and correlations with therapy response

In this cohort, most AI-treated tumors showed at least a moderate decline in TUV (mean decline 73.6%). Since AIs function by depleting the estrogen ligand without modifying the ER, this was not necessarily expected [28]. However, prior studies have also reported similar reduction in FES, as well as FDG, uptake after AI therapy in metastatic cohorts [40, 41], suggesting that ligand depletion via AI may induce tumor cellular dropout or down-regulate ER expression. Indeed, we initially hypothesized that reduced FES uptake would correlate with reduced tumor viability in the AI cohort. Our findings demonstrated a moderate, albeit non-significant, correlation between reduced FES uptake and reduced Ki-67. Notably, reductions in SUVpk and SULpk, key dbPET metrics representing the peak standard uptake value and such value relative to total lean body mass, respectively, positively correlated with reduced Ki-67 values. Nonetheless, the precise mechanisms underlying these findings are still unknown and require further studies to better understand what this may mean about tumor biology.

### SERM/SERD cohort: trends in FES uptake and correlations with therapy response

On the other hand, given the mechanism of action of SERM/SERD therapies, we hypothesized that incomplete reductions in FES uptake, thus reflecting inadequate tumor blockade, would correlate with poorer tumor response markers. Indeed, SUVpk and SULpk again showed strong positive correlations with changes in Ki-67 for the SERM/SERD cohort, with minimal reductions in FES uptake associated with minimal reductions in Ki-67 and MRI tumor longest diameter. These findings support the notion that residual FES uptake reflects inadequate receptor blockade [40]. Yet, the strong positive correlation we observed between residual FES signal and Ki-67 further suggests that this incomplete blockade may also be biologically meaningful, corresponding to reduced tumor suppression. Thus, in this SERM/SERD cohort, FES-dbPET may capture both pharmacodynamic effects and residual tumor viability.

### Correlations with MRI-based tumor size

Interestingly, change in FES-dbPET variables did not strongly correlate with MRI-based longest tumor diameter in either the AI or SERM/SERD cohort. This finding may reflect the pattern of tumor response to NET in ILC, with non-concentric shrinkage often being observed; further investigation into pathologic features such as tumor cellularity may be helpful in understanding this finding [42, 43]. When evaluating baseline FES-dbPET parameters, we noted the unexpected finding that while higher baseline SUVmean was associated with greater reduction in MRI-based tumor longest diameter, baseline TUV was negatively correlated with reduction in MRI-based tumor longest diameter. We hypothesize that baseline SUVmean may reflect treatment sensitivity to endocrine therapy, whereas TUV may represent a combination of both sensitivity to therapy and tumor size; greater volume of tumor at baseline might be associated with less tumor shrinkage. These intriguing findings raise the possibility that FES-dbPET may provide a more sensitive tool for detecting changes beyond what anatomical assessments from MRI can capture, with further study being needed.

### Clinical implications of findings

While similar studies assessing the utility of FES-dbPET in ILC patients based on therapy type remain very limited, a feasibility study conducted by Jones et al*.* also demonstrated the promising capability of FES-dbPET imaging in providing early predictions of neoadjuvant treatment efficacy and thereby helping to facilitate therapy selection [34]. Additionally, unlike FDG-PET, which often underperforms in ILC because of low tumor glycolytic activity, FES-dbPET directly quantifies functional ER with significant resolution, detecting heterogeneity that biopsies and MRI may miss [44]. Importantly, other molecular imaging modalities may be used to monitor response to neoadjuvant therapy, such as positron emission mammography (PEM), and whole body PET combined with either computed tomography or MRI. We studied dbPET for its theoretically higher resolution than whole body imaging; while PEM may prove to be a useful tool for monitoring treatment response, we do not have access to this imaging modality at our institution [45].

A 2025 meta-analysis of over 300 patients showed that baseline FES positivity on FES-PET triples the likelihood of endocrine benefit, strongly supporting the role of FES-PET as a successful biomarker for endocrine therapy success, largely in the metastatic setting [39]. Our work is the first to stratify that signal by drug class in the neoadjuvant, early-stage ILC setting with dbPET in particular. While current appropriate use criteria for FES suggest that using FES at diagnosis of early-stage breast cancer for consideration of endocrine therapy use or for measuring response to therapy is rarely appropriate, these findings suggest that serial FES during NET might help assess response and potentially guide treatment selection [26]. Validation of these pilot results could allow for the potential use of FES-dbPET in ILC as a non-invasive modality for response assessment during NET, which in turn could potentially help guide treatment duration or change.

Prior studies of FES-PET in the neoadjuvant setting have focused on predicting treatment response as a means of patient selection for NET versus neoadjuvant chemotherapy, or, to predict response to neoadjuvant therapy in patients with ER + HER2 + tumors. Chae et al. studied whole-body FES-PET in post-menopausal patients with ER + primary breast cancer randomized to either neoadjuvant chemotherapy (NAC) or NET on the NEOCENT trial [31]. In their analysis, higher baseline uptake of FES was not associated with differential response to either NAC or NET, while lower baseline uptake of FES appeared to be associated with improved response to NAC. Park et al. studied baseline FES-PET uptake in the primary tumor on whole-body imaging in 24 patients with ER + HER2 + tumors of no special type who received neoadjuvant therapy with aromatase inhibitor and a HER2 directed tyrosine kinase inhibitor [32]. In their study, lower baseline FES uptake was associated with reduced clinical response to the non-chemotherapy containing treatment regimen. Our study differs conceptually from both in that instead of aiming to utilize FES-dbPET parameters to identify patients who would benefit from NET, we instead sought to determine whether serial FES-dbPET parameters might reflect response to therapy as a pharmacodynamic and potentially prognostic marker, akin to change in the proliferation marker Ki67 as noted in the POETIC trial [16]. Current appropriate use criteria for FES in the early-stage setting suggest that FES is rarely appropriate for treatment selection; indeed, patients with ER positive tumors currently receive endocrine therapy regardless of tumor heterogeneity and even in the setting of ER-low status, as endocrine therapy is more tolerable than chemotherapy and is associated with improved survival even in the case of ER-low tumors [46]. For this reason, we aimed to focus on dynamic change in FES-dbPET after NET, as opposed to a static baseline value. This approach may result in greater clinical applicability to patients with ILC, in whom treatment response indicators are needed to help guide adjuvant treatment decisions.

### Limitations

Our study presents novel findings in the context of a prospective trial; however, it is important to acknowledge several limitations. First, the prospective design and logistical considerations posed challenges in obtaining baseline and post-treatment dbPET scans at consistent intervals for all patients. Delays in baseline FES-dbPET scans for some patients likely compromised the accuracy of our baseline measurements. Furthermore, we acknowledge that several patients underwent their post-treatment dbPET days to months before completing their full course of NET, introducing variability in the timing of post-treatment assessments. These factors likely led to an underestimation of the observed changes from pre-treatment to post-treatment scans. Additionally, the small sample size inherent in our prospective trial limited the statistical power of our analyses, underscoring the need to validate our results with larger clinical trials. However, the findings from this pilot study will inform ongoing, larger studies incorporating serial FES-dbPET in the neoadjuvant setting, such as the currently accruing I-SPY2 Endocrine Optimization Pilot study [33, 47]. Finally, we also acknowledge the potential limitations in using longest tumor diameter, as opposed to volumetric metrics, as an objective MRI-based tumor measurement. However, given the clinical use of longest tumor diameter for surgical planning, we opted to use this more clinically relevant measurement technique in our study.

## Conclusion 

In this prospective pilot study of 19 patients with early stage ILC, serial FES-dbPET showed capability for detecting ILC tumors and demonstrated correlations with markers to tumor response to NET, potentially offering valuable insights into tumor biology beyond what is captured by standard imaging metrics. Notably, changes in SUVpk and SULpk from dbPET were correlated with changes in Ki-67, highlighting the potential of FES-dbPET parameters as non-invasive biomarkers of treatment response. These results demonstrate the potential of this novel imaging tool for evaluating treatment response in the pre-operative setting. Given the unmet need for improved imaging tools in ILC, further research is warranted to better understand the significance of FES-dbPET features and refine their application in enhancing neoadjuvant treatment monitoring and surgical planning.

## Supplementary Information

Supplementary Material 1.

## Data Availability

The datasets generated during and/or analyzed during the current study are not publicly available due to risk of compromising individual privacy but are available from the corresponding author on reasonable request.
