# Supplementary figures and images for: A prospective single-arm pilot study evaluating [F-18]fluoroestradiol dedicated breast PET in invasive lobular carcinoma after neoadjuvant endocrine therapy
Source: Breast Cancer Res. 2026 Apr 11;28:93. doi: 10.1186/s13058-026-02277-w (PMC13185200; doi:10.1186/s13058-026-02277-w)

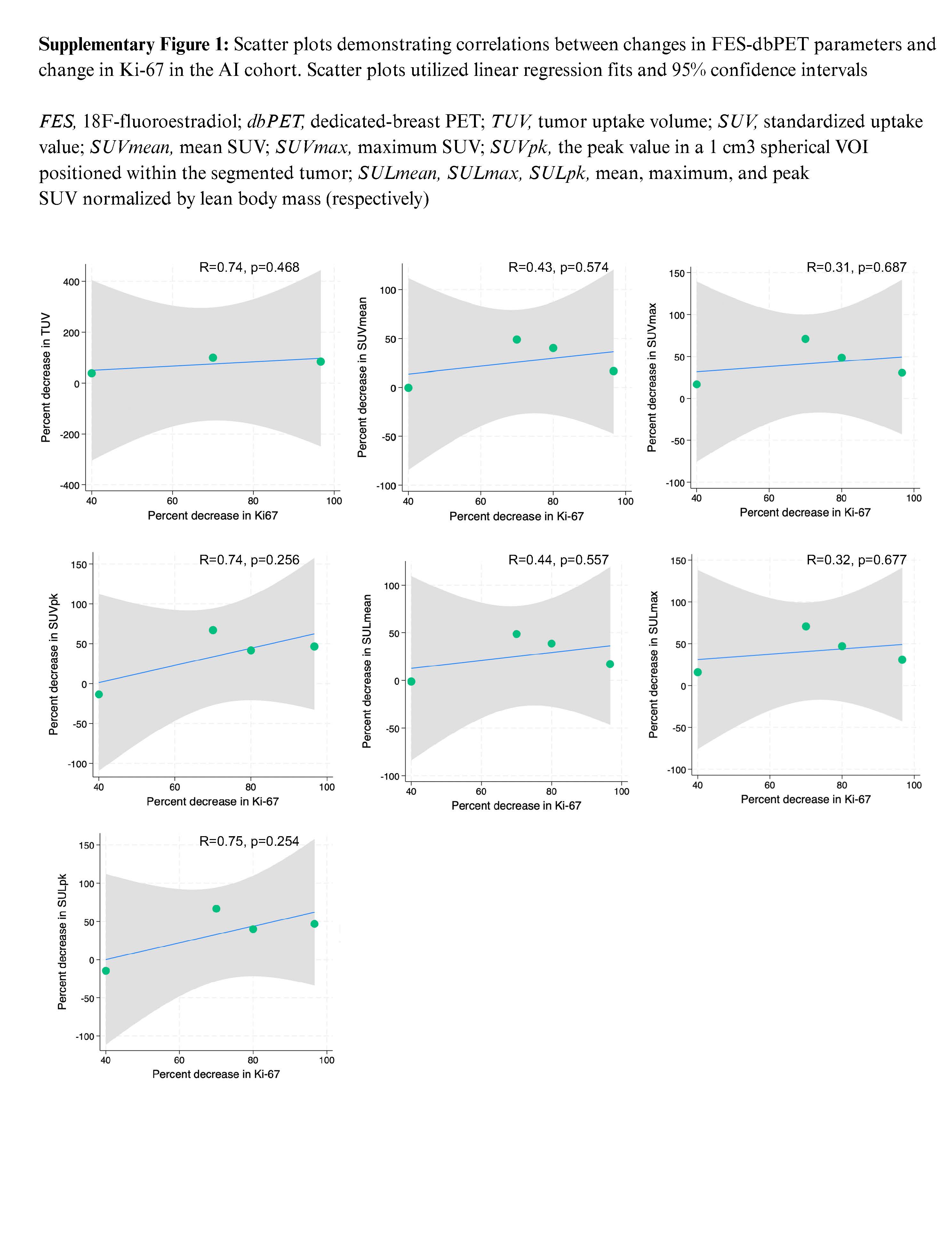

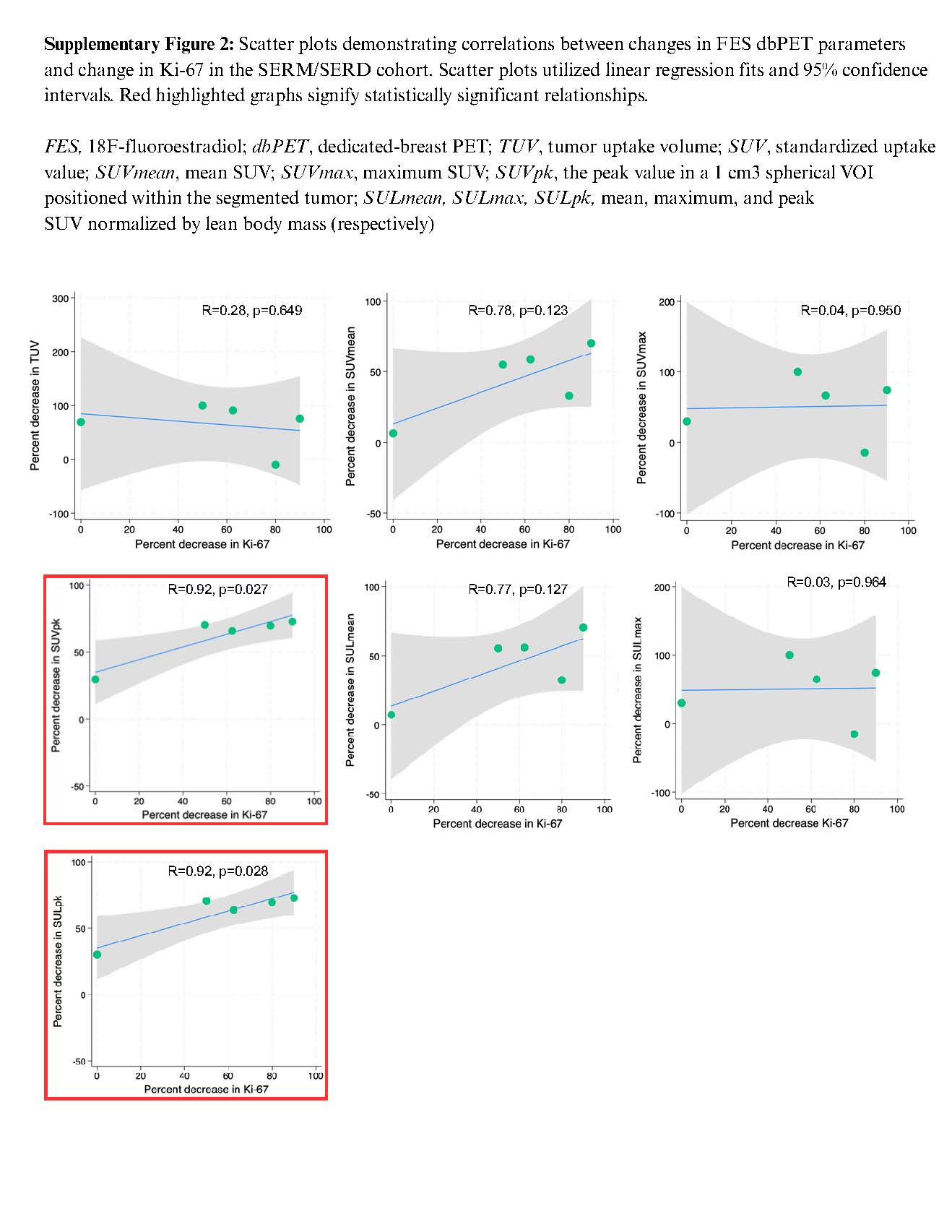

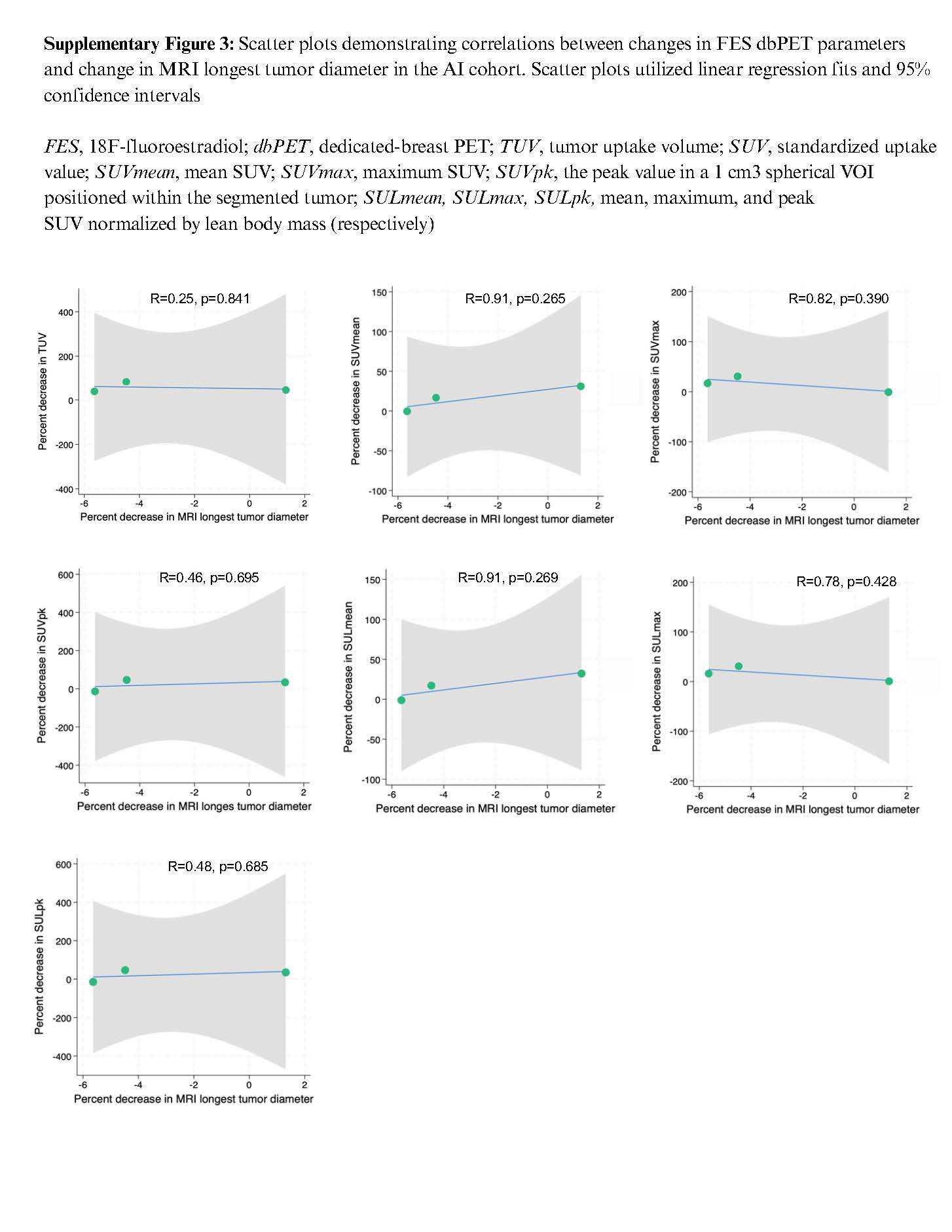

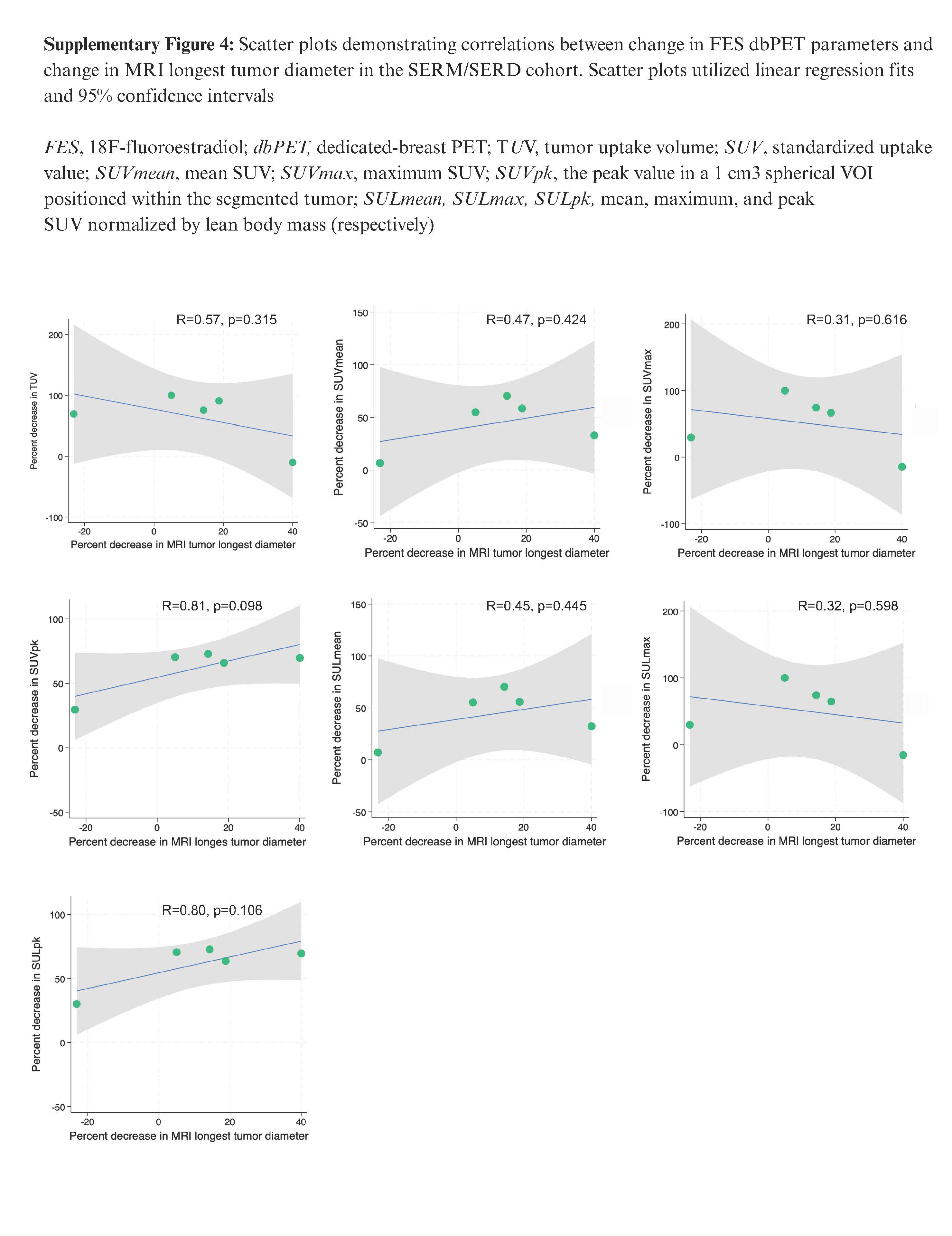

Supplement: Supplementary file 1 — Supplementary Material 1. [file 13058_2026_2277_MOESM1_ESM.docx]
